# Reactive aldehyde species–mediated neuropathic pain in spinal cord injury: Mechanisms and therapeutic opportunities

**DOI:** 10.4103/NRR.NRR-D-25-00126

**Published:** 2025-09-03

**Authors:** Rachel L. Stingel, Nicholas S. Race, Riyi Shi

**Affiliations:** 1 Weldon School of Biomedical Engineering, Purdue University, West Lafayette, IN, USA; 2 Department of Basic Medical Sciences, College of Veterinary Medicine, Purdue University, West Lafayette, IN, USA; 3 Center for Paralysis Research, Purdue University, West Lafayette, IN, USA; 4 Department of Physical Medicine & Rehabilitation, University of California Davis, Sacramento, CA, USA; 5 Safar Center for Resuscitation Research, Pittsburgh, PA, USA; 6 Association of Academic Physiatrists Rehabilitation Medicine Scientist Training Program, Owings Mills, MD, USA

**Keywords:** acrolein, inflammation, neuropathic pain, reactive aldehyde species, spinal cord injury

## Abstract

Spinal cord injury–related neuropathic pain is difficult to treat and significantly decreases quality of life. Often functionally limiting and refractory to conventional pharmacologic treatments, treating spinal cord injury–related neuropathic pain necessitates the exploration of novel mechanistic therapeutic strategies. Mounting preclinical and clinical evidence suggests that neurotoxic reactive aldehyde species such as acrolein, produced endogenously in high concentrations following spinal cord injury, are putative therapeutic targets in spinal cord injury–related neuropathic pain. The purpose of this review is to synthesize the current knowledge of mechanisms underlying reactive aldehyde species–mediated pain following spinal cord injury and propose novel drug strategies for future investigation. In doing so, we aim to highlight emerging evidence that supports the use of interventions designed to decrease reactive aldehyde species and their potential to mitigate the detrimental impacts of neuropathic pain in spinal cord injury. Ultimately, reactive aldehyde species–targeted therapeutics may have implications beyond spinal cord injury, as reactive aldehyde species play a pivotal role in the pathology of many neurological disorders.

## Introduction

Spinal cord injury (SCI) is caused by an initial insult to the spinal cord (primary injury) and subsequently triggers a series of complex pathological events (secondary injury), resulting in profound functional loss. The most common cause of SCI is mechanical trauma (e.g., motor vehicle accidents and falls), but it can also arise from ischemia, bleeding, infection, inflammation, or neoplasms. In most cases, neurological function below the level of injury is substantially impaired, resulting in long-term sensory, motor, visceral, and autonomic dysfunction. Neuropathic pain (NP) is another complication estimated to affect approximately half of all individuals with SCI (Burke et al., 2017). SCI-related NP (SCINP) results in decreased functional abilities and productivity, increased healthcare utilization/costs (Margolis et al., 2014), and an increased risk of comorbidities (Mann et al., 2013). Psychological implications associated with SCINP include decreased mood, sleep, functional capacity, and relationship quality (Migliorini et al., 2008; Hoffman et al., 2011; Mann et al., 2013; Cuff et al., 2014; Shiao and Lee-Kubli, 2018).

SCI is considered rare, affecting approximately 294,000 individuals in the United States with approximately 17,000 new cases annually (National Spinal Cord Injury Statistical Center, 2020; World Health Organization, 2024); however, the economic burden of SCI and SCINP is disproportionately large. In 2014, the average annual cost to manage NP was estimated to be $27,259 per person in the United States (Schaefer et al., 2014), while the overall cost of living with SCI ranges from $45,000 to 1.5 million per year (National Spinal Cord Injury Statistical Center, 2020). Those with SCINP are estimated to incur an average of more than $26,000 in additional healthcare costs annually alongside increased healthcare resource utilization (Mann et al., 2013) compared to SCI patients without NP (Mann et al., 2013). Despite its prevalence, economic burden, and high incidence of associated disability, SCINP is often refractory to available treatments. Three-quarters of individuals with SCINP report inadequate pain control despite spending an average of $4,300 per year on medications and chronically taking an average of at least two for pain, which often include opioids (Cardenas and Jensen, 2006; Mann et al., 2013). The difficulty of treating SCINP is multifactorial and includes an incomplete understanding of the underlying mechanisms, inadequate animal models, pain heterogeneity, symptomatic treatment strategies, and non-specific or undruggable treatment targets.

Neurotoxic reactive aldehyde species (RAS) produced during secondary injury cascades, and their downstream receptor/channel-mediated actions, have recently been identified in mechanisms underlying and as potential therapeutic drug targets for SCINP (Park et al., 2014b, 2015; Chen et al., 2016b; Lin et al., 2018; Herr et al., 2021). The purpose of this review is to discuss the current scientific understanding of SCINP, and SCINP-relevant pathological consequences of RAS, and to provide insights into a novel class of potential therapeutic strategies focused on decreasing RAS levels to provide symptomatic relief with disease-modifying potential in SCINP.

## Search Strategy

This narrative review utilized an unstructured search strategy guided primarily by the authors’ expertise and experience in this scientific domain augmented by careful review of the literature. Databases utilized to identify relevant articles included PubMed and Google Scholar. Relevant keyword combinations utilized to explore major themes in the manuscript included: “Spinal Cord Injury AND Neuropathic Pain,” “Spinal Cord Injury AND Reactive Aldehyde Species,” “Neuropathic Pain AND Reactive Aldehyde Species,” “Reactive Aldehyde Species AND Transient Receptor Potential Ankyrin.” Focused, topical searches also included keyword combinations involving the aforementioned phrases in addition to: “Inflammation,” “Mitochondrial Dysfunction,” and “Carbonylation.” All study years were included.

## Clinical Spinal Cord Injury–related Neuropathic Pain and Current Therapeutic Strategies

NP is often described as a burning, stabbing, or tingling sensation that is either spontaneous or evoked by exogenous stimuli such as touch or temperature. Increased responsiveness occurs when non-painful stimuli are perceived as painful (allodynia), or when painful stimuli are more painful than expected (hyperalgesia). The pathophysiology underlying these symptoms includes a phenomenon termed central sensitization, which describes progressively centralizing somatotopic reorganization that intensifies spontaneous pain sensations and/or evoked responsiveness to stimuli.

NP, SCI, and SCINP are difficult conditions to characterize (Barbee Jr et al., 2018; Finnerup et al., 2021). For NP, various scoring systems have been developed as qualitative research tools, but are not widely used in clinical settings. Evoked potentials provide an avenue for quantitative assessment, but are technically challenging, personnel-intensive, and not widely used for research or clinical study of NP. Practically, NP is delineated into central and peripheral pain and further characterized by the location of damage (Treede et al., 2008; Finnerup et al., 2016, 2021). In instances of neuronal injury, direct damage to the central nervous system (CNS) and subsequent pain is referred to as central pain, whereas pain radiating from damaged nerve roots or peripheral nerves is termed peripheral pain (Scholz et al., 2019; Finnerup et al., 2021). SCINP is further subclassified by the location of pain in relation to the injury. “At-level pain” is pain that usually develops shortly after injury and arises from damaged nerve roots at or around the level of SCI, while “below-level pain” can take weeks to years to develop and arises from secondary inflammatory, degenerative damage to ascending sensory tracts, or changes in sensory processing below the level of injury (Hulsebosch et al., 2009; Lee et al., 2013; Shiao and Lee-Kubli, 2018). Standardized neurological exams, namely the widely accepted American Spinal Injury Association Impairment (ASIA) Scale, are used to assess the extent of sensory and motor loss from SCI and predict the extent of recovery in a mobility-focused manner (Roberts et al., 2017). The ASIA scale characterizes the sensory and motor function below the level of injury as complete or to varying degrees of incomplete. The ASIA scale has been updated multiple times to reflect advancements in diagnostic capabilities and to improve its reliability (Roberts et al., 2017; Alizadeh et al., 2019). Nonetheless, pain, among other critical domains, is not a factor included in the ASIA assessment despite profound functional implications.

Pain relief is one of the highest-rated priorities by individuals with SCI (Henwood and Ellis, 2004; Bloemen-Vrencken et al., 2005; Kennedy et al., 2006; Simpson et al., 2012). All current treatments for SCINP are symptomatic rather than mechanistic or disease-modifying and existing drugs have significant shortcomings and side effects (**Table 1**). First-line pregabalin, the only drug specifically US Food and Drug Administration-approved for SCINP, and gabapentin have abuse potential and can cause drowsiness, dizziness, impaired coordination, increased fall/injury risk, and are associated with higher risk for suicide and unintentional overdose (Toth et al., 2009; Shanthanna et al., 2017; Molero et al., 2019). Second-line antidepressants have not demonstrated direct efficacy for SCINP (Hagen and Rekand, 2015). Tricyclic antidepressants can cause dry mouth, constipation, blurred vision, sedation, confusion, and urinary retention, with a narrow therapeutic index and risk of overdose from fatal arrhythmias (Trindade et al., 1998; Finnerup et al., 2015). Serotonin-norepinephrine reuptake inhibitors can cause nausea, drowsiness, gastrointestinal issues, and tachycardia (Stahl et al., 2005). Opioids are addictive and can lead to tolerance, physical dependence, constipation, respiratory depression, and an increased risk of overdose and death (Kalso et al., 2004; Moore and McQuay, 2005; Finnerup et al., 2015; Rudd et al., 2016). Many of these side effects are also direct consequences of SCI itself (Brown et al., 2006; Sezer et al., 2015), increasing the risk of adverse health events related to such therapeutics. Comorbid anxiety and depression occur at ≥ 2× the general population rates in SCI patients, further increasing the risk of opioid misuse, abuse, addiction, and opioid-related death (Brooner et al., 1997; Sullivan et al., 2006; Migliorini et al., 2008; Webster et al., 2011). Patients failed by medications often pursue invasive procedural interventions, which bear inherent risk and lack substantive evidence of efficacy (Kumar et al., 2007, 2008; Van Calenbergh et al., 2009; Cho et al., 2015; Wu et al., 2018).

**Table 1:** Current standards of care for clinical SCINP

Drug	First agent prescribed	Mechanism of action	Efficacy	Side effects
Pregabalin (Lyrica)	20.3%	Inhibits voltage-gated N-type calcium ion channels at the α2δ subunit	Moderate-to-strong effect sizes (0.695–3.805) in 3 SCINP studies In non-SCINP, NNT* 6.5–9.4 First line treatment	Somnolence, dizziness, peripheral edema, asthenia, dry mouth, constipation
Gabapentin (Neurontin)	50.1%		Strong effect sizes (0.873–3.362) in 4 of 6 SCINP studies NNT 5.9–9.1 in non-SCINP First line treatment	Somnolence, dizziness, diarrhea, constipation, peripheral edema, asthenia, weight gain, dry mouth
Amitriptyline (Elavil, Endep)	5.6%	Nonselective noradrenaline and serotonin reuptake inhibition	Inconclusive SCINP efficacy (no effect in one study) In non-SCINP, NNT 3–4.5; may work best in patients with depression Second line treatment	Dry mouth, orthostatic hypotension, constipation
Duloxetine (Cymbalta)	8.9%	Selective noradrenaline and serotonin reuptake inhibition	No direct evidence in SCINP; in non-SCINP, NNT 5.2–8.4 may work best in patients with depression Second line treatment	Somnolence, nausea, vomiting, dizziness, confusion, headache
Venlafaxine (Effexor)	4.1%			Nausea, headache, sedation, dizziness
Opioids short-acting	78.4% co-treatment	μ-opioid receptor agonism	Limited efficacy in SCINP in non-SCINP, NNT 3.4–5.8 Third-line or co-treatment	Somnolence, dry mouth, dizziness, sweating, constipation, nausea, respiratory depression, dependence
Opioids long-acting	23.8% co-treatment			

* NNT refers to number of patients treated for one patient to have clinically meaningful reduction of pain. SCINP: Spinal cord injury-related neuropathic pain.

To our knowledge, all current and in-development therapies (15 active clinical trials) for SCINP are symptomatic treatments. A single new chemical entity investigated as a disease-modifying intervention in SCINP, KAI-1678, has recently failed to demonstrate efficacy in Phase II trials, consequently and indefinitely halting its development. Several other clinical trials assessing the analgesic potential of cannabinoids are also underway although evidence from recent systematic reviews does not support their efficacy in SCINP (Hagen and Rekand, 2015). The failure of currently available treatments underscores a pressing need for novel, mechanistic strategies that target underlying causes rather than resultant symptoms.

## Reactive Aldehyde Species and Related Mechanisms in Spinal Cord Injury–related Neuropathic Pain

### Overview of spinal cord injury–related neuropathic pain pathobiology

SCINP can arise from primary and/or secondary SCI. The primary injury results in immediate cell death at the injury site (Crowe et al., 1997), blood–spinal cord barrier disruption, hypoxia, ischemia related to vasospasm (Noble and Wrathall, 1988; Tator and Fehlings, 1991; Figley et al., 2014), and neural circuit destruction (Woolf and Costigan, 1999; Woolf and Salter, 2000; Kawasaki et al., 2008; Tan and Waxman, 2012; Ji et al., 2016). Importantly, a previous study has shown that an isolated focal spinal nerve injury produced by ligation is capable of recapitulating NP symptoms (Ho Kim and Mo Chung, 1992). Given the often more extensive damage from the primary injury alone, it is reasonable to conclude that such an event may lead to similar, if not more severe, and mechanistically complex NP.

The secondary injury begins within minutes of the primary injury and further perpetuates and worsens the extent of damage (Hausmann, 2003). The secondary injury can be broken into three phases and the mechanisms that compose these phases can persist for seconds to years (Hausmann, 2003; Alizadeh et al., 2019; Bradbury and Burnside, 2019). The acute stage (0–48 hours post-SCI) is marked by immune cell infiltration, ionic imbalances, excitotoxicity, reactive species formation, mitochondrial dysfunction, demyelination, and mixed necrotic/apoptotic cell death (Hausmann, 2003; Alizadeh et al., 2019; Anjum et al., 2020). The sub-acute phase (48 hours–2 weeks) is characterized by pathological demyelination, axonal degeneration, debris accumulation, an ongoing inflammatory response, and further apoptosis (Hausmann, 2003; Alizadeh et al., 2019; Anjum et al., 2020). It is at this point that recovery mechanisms such as neuroplasticity, circuit reorganization, and signaling changes begin, albeit with limited success partially due to the toxic peri-injury milieu, which includes RAS and activated inflammatory cells, among other mediators (e.g., reactive oxygen and nitrogen species, reactive oxygen species (ROS) and reactive nitrogen species, respectively) (Nagappan et al., 2020). During the chronic phase (> 2 weeks post-SCI), repair efforts cease due to glial scar formation (Bradbury and Burnside, 2019). At this point, further functional restoration depends on neuronal plasticity and functional re-training.

Ultimately, SCI disrupts the integrity of healthy sensory neural transmission and neuronal architecture, which can collectively disrupt pain modulation and reduce the ability to curtail the excitation of nociceptive neurons (Finnerup et al., 2021). Chronic nociceptor (Bedi et al., 2010; Ritter et al., 2015), and dorsal horn superficial laminar neuron (Hains et al., 2003; Hains and Waxman, 2006; Latremoliere and Woolf, 2009) hyperactivity is exacerbated by hypersensitivity and leads to secondary plastic changes in spinal cord and brain circuits involved in nociceptive processing, thereby leading to enhanced stimulus transmission to the CNS and contributing to central sensitization (Baron, 2006).

### Reactive aldehyde species: Sources, metabolism, and biological targets

**Figure 1:**
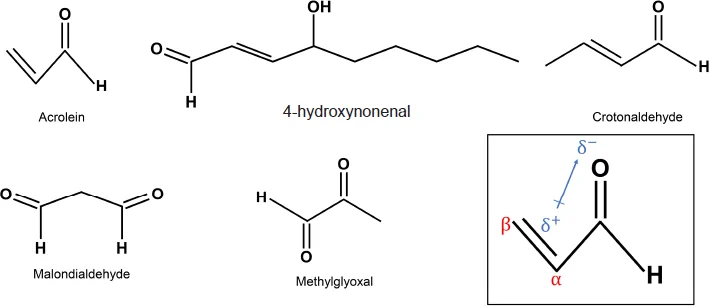
Structures of common endogenous reactive aldehyde species produced from lipid peroxidation and carbohydrate metabolism (methylglyoxal). Reactive aldehyde species are electrophilic compounds characterized by a center carbonyl flanked by an R group and hydrogen. Electrophile reactivity is determined by the strength of electron-withdrawing groups. Regarding α,β unsaturated aldehydes (acrolein and 4-hydroxynonenal), the carbonyl draws electrons away from the carbon double bond, creating an electron-poor area that is susceptible to attack from electron-rich biomolecules, as depicted by the boxed acrolein on the bottom right.

Neurotoxic small molecules known as RAS (**Figure 1**) are endogenously produced at high rates in response to neurological insults. In an animal model of SCI, Kaynar et al. (1998) observed malondialdehyde increases alongside injury severity, demonstrating a correlational link between RAS levels and SCI outcomes; we have observed similar severity-dependent post-injury elevations of acrolein in our lab (Luo et al., 2005; Park et al., 2014b, 2015). RAS levels are highest in the acute secondary injury, with increases beginning within 1-hour post-injury. Although levels gradually decrease towards the chronic stage, RAS levels remain elevated in spinal cord peri-lesional tissue, serum, and urine of SCI-injured rats for at least 2 weeks post-injury (Park et al., 2014b, 2015; Chen et al., 2016b; Tian and Shi, 2017). In lab-generated (guinea pig, rat) and naturally-occurring SCI (dog) (Sangster et al., 2017), acrolein levels can reach 700–1100 ng/mg (40–100 ng/mg in controls) (Due et al., 2014; Park et al., 2014b, 2015; Chen et al., 2016b; Tian and Shi, 2017). Concentrations in this range are capable of damaging mitochondria, cells, and *ex vivo* spinal cords (Luo and Shi, 2005).

Although RAS are generally produced during oxidative stress, a condition in which excessive oxygen-free radicals are produced and ineffectively cleared, it is also the byproduct of other enzyme-mediated reactions (e.g., glycolysis, ethanol metabolism) (Kuykendall, 2010; Allaman et al., 2015). Oxidative stress is a response to direct spinal cord damage and is sustained through the acute and sub-acute secondary injury periods (Panter et al., 1990; Dawson et al., 1993; Facchinetti et al., 1998; Liu et al., 1998; Luo et al., 2002; Luo and Shi, 2004, 2005; Bains and Hall, 2012; Wells et al., 2012; O’Hare Doig et al., 2014, 2017; Milbourn et al., 2017). Free radicals are produced immediately following SCI (Liu et al., 1998) and notably activate neutrophils, causing them to produce RAS through myeloperoxidase-catalyzed degradation of threonine and indirectly activate leukocyte-mediated pro-inflammatory signaling mechanisms (Anderson et al., 1997).

**Figure 2:**
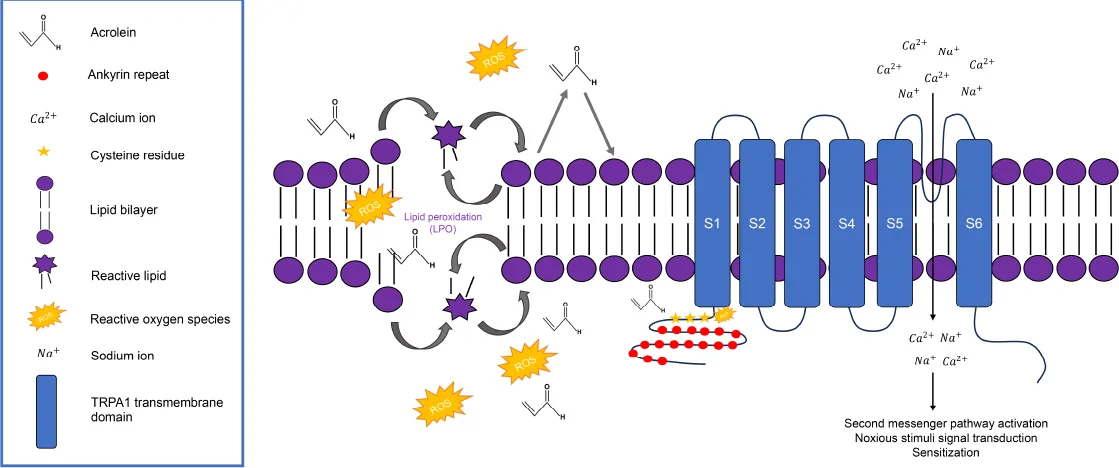
TRPA1-induced activation through acrolein binding. TRPA1 is a non-selective cation channel composed of six transmembrane domains, with a pore-loop between the 5^th^ (S5) and 6^th^ (S6) subunits. Specific to only TRPA1, the N-terminal contains ankyrin repeats, which no other member of the TRP channel family has. RAS (acrolein) produced from oxidative stress-induced LPO activates TRPA1 by binding to cysteines on the N-terminal. TRPA1 activation enables cation (Ca^2+^ and Na^+^) influx, pain signal transduction, and second messenger activation. Modified from Chen and Hackos (2015). LPO: Lipid peroxidation; RAS: reactive aldehyde species; TRPA1: TRP ankyrin 1.

Several additional RAS production pathways are activated during the acute stage in response to the microenvironment including amine oxidase and polyamine oxidase-mediated polyamine catabolism, carbohydrate autooxidation under hyperglycemic conditions, and lipid peroxidation (LPO) (Esterbauer et al., 1991; Stevens and Maier, 2008). Oxidative stress–induced LPO produces RAS byproducts such as 4-hydroxynonenal (4-HNE), malondialdehyde, and acrolein (**Figures 1** and **2**) (Luo and Shi, 2004; Shi et al., 2011a, 2015; Due et al., 2014; Park et al., 2015; Butler et al., 2017). In LPO, free radicals oxidize polyunsaturated lipids, inducing a chain reaction capable of destroying cell membranes and myelin (**Figure 2**). This results in axonal degeneration, demyelination, cellular signaling disruption, and cell death (Shi et al., 2002; Peasley and Shi, 2003; Luo and Shi, 2004, 2005; Liu-Snyder et al., 2006b; Sun et al., 2012; Ravera et al., 2015). RAS notably induce a feed-forward effect, wherein elevated RAS concentrations lead to further RAS production, ROS production, and LPO (**Figure 2**), inducing a vicious cycle that can overwhelm the body’s endogenous neutralization/elimination capacities.

Neurons in the peripheral nervous system (PNS) and CNS are highly susceptible to oxidative stress, LPO, and RAS due to their high content of membrane phospholipids, axoplasmic mitochondria, and weak antioxidant defenses (Low et al., 1997; Chen et al., 2012; Areti et al., 2014; Carrasco et al., 2018). Neuronal antioxidant defenses are easily overwhelmed in conditions with significant cellular stress such as direct trauma or chronic inflammation, both of which occur during secondary injury. Glutathione (GSH) is an important endogenous antioxidant that neutralizes RAS via reduction reactions. RAS-GSH adducts are further metabolized into safe byproducts by enzymes in the aldehyde dehydrogenase (ALDH) superfamily. However, RAS can exacerbate oxidative stress by depleting GSH levels more rapidly than GSH can be produced, and depleted GSH levels lead to redox imbalances, resulting in LPO and mitochondrial dysfunction (Mitchell and Petersen, 1989; Stevens and Maier, 2008; Shi et al., 2011a). RAS-depleted GSH stores have also been linked to glutamate excitotoxicity via the Xc exchange (cysteine/glutamate antiporter) system. Here, GSH precursor cysteine is exchanged for glutamate, which is then released extracellularly (Barger et al., 2007). Glutamate excitotoxicity is thought to be a fundamental mechanism in the secondary injury that can lead to ROS formation, inflammation, and cellular hyperexcitability (reviewed in Hulsebosch et al., 2009; Carrasco et al., 2018).

The downstream effects of oxidative stress and LPO include DNA and protein modification/damage, inflammatory transcription factor activation/production (reviewed in Chen et al., 2012), activation/dysregulation of cell proliferation, signaling, and cell death pathways (O’Hare Doig et al., 2014, 2017; Li et al., 2022). Furthermore, oxidative stress induced by traumatic brain and SCI can damage the blood–brain barrier and blood–spinal cord barrier (Baldwin et al., 1998; Abdul-Muneer et al., 2013), suggesting such mechanisms may facilitate peripheral immune cell migration into the immune-privileged CNS. The subsequent subacute and chronic inflammatory milieu may also perpetuate oxidative stress, as macrophages and neutrophils are known to generate RAS following SCI and other inflammatory states (Carlson et al., 1998; Kirkham et al., 2003; Malcangio, 2019). Sustained oxidative stress has been heavily implicated in the mechanisms contributing to NP (reviewed by Liu et al., 1998; Chen et al., 2012; Fritz and Petersen, 2013; Hassler et al., 2014; Li et al., 2022).

Beyond direct tissue destruction, redox dysregulation, and pro-inflammatory signaling, RAS can also bind directly to proteins and enzymes to cause damage and dysfunction (Arumugam et al., 1999; Luo and Shi, 2004, 2005; Chavez et al., 2011; Hill et al., 2018). RAS are extremely reactive electrophiles, owing to their steric and electric properties stemming from their common α,β-unsaturated carbonyl (**Figure 1**). These inherent properties allow RAS to bind to and covalently modify proteins and DNA at nucleophilic sites (Esterbauer et al., 1991; LoPachin et al., 2009; Chavez et al., 2011; Fritz and Petersen, 2013; Spickett, 2013; Aslebagh et al., 2016). RAS preferentially form adducts with thiol (sulfhydryl, -SH) groups on cysteine residues (LoPachin et al., 2007, 2009; Cai et al., 2009) although they can react with other amino acids such as histidine and lysine moieties (Uchida et al., 1998; Chen, 2023). Cysteine residues are nucleophiles present in high concentrations on mitochondrial proteins and transmembrane proteins, e.g., TRP ankyrin 1 (TRPA1).

**Figure 3:**
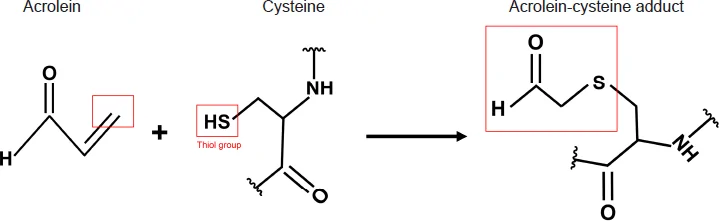
Acrolein-cysteine residue Michael addition. Electrophilic reactive aldehyde species are highly reactive towards cysteine residues, including the ones that modulate TRP ankyrin 1 channels. This schematic depicts the adduct formed between the β-carbon on acrolein and the thiol group on cysteine. As a result of adduct formation, the cysteine residues form a disulfide bridge, which leads to a conformational change of the N-terminus and opens the channel. Interestingly, cysteine residues can act as redox sensors by reversibly undergoing oxidation and reduction in response to environmental redox changes (Bessac et al., 2008; LoPachin et al., 2009).

Adduct formation can occur through Michael Addition (**Figure 3**), where the β-carbon of the RAS reacts with a nucleophile (e.g., thiol group on cysteine), resulting in a 1,4-addition or Schiff base reaction, where the carbonyl carbon on the RAS reacts with primary amines (e.g., lysine) via 1,2-addition (Ishii et al., 2007; Cai et al., 2009; LoPachin et al., 2009). Whereas Schiff base reaction adducts are typically unstable intermediates and thus reversible, Michael addition products can cause permanent protein modifications with significant ramifications on cellular function (e.g., enzyme, mitochondrial, or cell signaling disruption) (LoPachin et al., 2009). For example, RAS likely bind to and impair the action of adenine nucleotide translocase (Luo and Shi, 2005) and α-ketoglutarate dehydrogenase (Klingenberg and Nelson, 1994; Humphries et al., 1998), exacerbating oxidative stress (Esposito et al., 1999; Wallace, 1999, 2001; Luo and Shi, 2005; Shi et al., 2011a). RAS can also overwhelm mitochondrial aldehyde dehydrogenase 2 (ALDH2), which can perpetuate oxidative stress, as observed in an animal model of SCI (Herr et al., 2021).

The formation of RAS-thiol adducts through Michael addition creates products with higher reactivity than free RAS (Cai et al., 2009). As a result, these adducts can independently form Schiff base adducts with other nearby targets (Cai et al., 2009). Similarly, multiple RAS can react with the same nucleophilic residue to form more complex structures, which may play a role in the formation of protein aggregates seen in some neurodegenerative diseases. Our group and others have observed RAS-mediated alpha-synuclein (Wang et al., 2017; Ambaw et al., 2018; Acosta et al., 2019) and amyloid-β (Rogers, 2023; Rogers et al., 2023) aggregations, which have relevance for Parkinson’s and Alzheimer’s diseases, respectively. The formation of RAS adducts prolongs their biological half-lives (Esterbauer et al., 1991; Jian et al., 2007; LoPachin et al., 2009; Fritz and Petersen, 2013; Moghe et al., 2015; Sánchez-Palencia et al., 2017), a feature that both extends the duration of their pathological impact and makes drug targeting more feasible than ROS/reactive nitrogen species, which have shorter half-lives. RAS and their adducts can also travel intra- and extra-cellularly to sites distant from their production (Ghilarducci and Tjeerdema, 1995; Luo et al., 2005; reviewed by Esterbauer et al., 1991; Shi et al., 2011a; Fritz and Petersen, 2013), including between the CNS and PNS. As such, RAS could be produced in the CNS from SCI, but diffuse to PNS loci and as a result achieve multiple-locus activation of pain signaling pathways.

### Reactive aldehyde species–induced mechanisms of spinal cord injury–related neuropathic pain

#### Overview of reactive aldehyde species in spinal cord injury–related neuropathic pain

Following SCI, RAS synergistically activate and upregulate important pain receptors and incite parallel neuroinflammatory pathways leading to behavioral hypersensitivity, chronic hyperexcitability, and culminate in chronic hypersensitization of the pain neuraxis. Indeed, RAS load has been correlated with behavioral hypersensitivity in rodent models across multiple studies (Patwardhan et al., 2010; Riahi et al., 2010; Zambelli et al., 2014). Acrolein alone can induce motor deficits reminiscent of SCI, SCINP-like hypersensitivity to noxious stimuli, and histologically recapitulates demyelinating and inflammatory neuropathology as demonstrated via injection into healthy rat spinal cords (Due et al., 2014; Park et al., 2014b, 2015; Gianaris et al., 2016). RAS elevations are thus temporally associated with and independently capable of reproducing key histopathological findings and behavioral phenotypes observed in SCI animal models. Mechanistic work has further demonstrated that RAS effects on key protein targets related to pain and neuroinflammation likely underpin, at least in part, post-injury development of SCINP-like behavioral hypersensitivity in animal models.

#### Post-translational modification of pain-relevant membrane proteins

Many effects exerted by RAS occur via covalent protein modification, resulting in altered structure-function relationships. One family of proteins, transient receptor potential (TRP) channels, are of particular interest in SCINP because of their involvement in chemical sensing, cytokine release, inflammatory regulation, and pain signal transduction to the CNS (Clapham, 2003; Wang and Woolf, 2005; Silverman et al., 2020). The TRP family contains numerous polymodal cation channels expressed ubiquitously throughout the body (Anand et al., 2008; Atoyan et al., 2009; Uta et al., 2010; Garrison and Stucky, 2011; Choi et al., 2014). Channels in this family are composed of six transmembrane domains and cytoplasmic amino and carboxy termini (Patapoutian et al., 2009; Garrison and Stucky, 2011; **Figure 2**). Key pain-relevant TRP family members include TRP vanilloid 1 (TRPV1) and TRPA1, which have been strongly implicated in inflammatory (Bautista et al., 2006) and neuropathic pain (Wang and Woolf, 2005). Peripherally, TRPA1 and TRPV1 are primarily found on unmyelinated C-fibers, and thinly myelinated Aδ-fibers (Uta et al., 2010) and co-localize on 30%–50% of nociceptive neurons (Bautista et al., 2006). TRPA1 is expressed on peripheral sensory nerve terminals, neuronal cell bodies and axons in dorsal root ganglia (DRG), the spinal cord dorsal horn, and numerous brain regions, as well as infiltrating peripheral macrophagies, resident glial cells (e.g. astrocytes, oligodendrocytes, microglia), and cerebrovascular endothelium (Lee et al., 2016; Kheradpezhouh et al., 2017; Xia et al., 2019; Naert et al., 2021).

TRPA1 and TRPV1 sensitivity may underlie neuronal hyperresponsiveness or low activation thresholds observed in allodynic (evoked) NP. Further, their tonic or constitutive activation via post-translational modifications may lead to spontaneous NP. The widespread expression of these receptors provides a mechanistic link for central sensitization involving numerous cell types and critical locations along the pain neuraxis from the PNS and CNS. Notably, the local application of a TRPA1 antagonist directly into the amygdala had no impact on pain thresholds in healthy animals, but it was analgesic in animals with NP (Sagalajev et al., 2018). In support of the idea that central sensitization and central expression of TRPA1 in the brain is an important mechanism in SCINP, it is worth noting that work in inflammatory and neuropathic pain models has observed that peripherally-restricted TRPA1 antagonists require higher doses for analgesic efficacy compared to antagonists that can cross the blood–brain barrier (Mesch et al., 2023).

TRPA1 can be activated by exogenous mechanical, thermal, and chemical stimuli, or via endogenous stimuli such as RAS; activation leads to inflammation, ROS formation, and downstream inflammatory gene expression (Basbaum et al., 2009; Choi et al., 2014; Achanta and Jordt, 2017). Endogenous activation and modulation of TRPA1 typically occur through covalent modifications of its amino acid residues, specifically cysteines on its N-terminus. Importantly, electrophilic compounds produced by oxidative stress such as ROS, RAS, and LPO products have a high affinity for the thiol groups on cysteines (Cai et al., 2009). Thus, oxidative stress byproducts produced following SCI can bind to and covalently modify cysteine residues on the N-terminus of TRPA1 (Hinman et al., 2006; Macpherson et al., 2007; Takahashi et al., 2008; Samanta et al., 2018). The cysteine residues form a disulfide bridge, inducing a conformational change of the N-terminus that opens TRPA1, which permits cation (calcium [Ca^2+^] and sodium [Na^+^]) influx, nociceptor depolarization and excitation and/or second messenger signaling activation (Basbaum et al., 2009; Choi et al., 2014; Achanta and Jordt, 2017; **Figure 2**). TRPA1-induced neuronal depolarization causes the release of calcitonin gene-related peptide, substance P, adenosine triphosphate, and glutamate onto superficial dorsal horn neurons. When N-methyl-D-aspartate receptors in the dorsal horn are activated, cation influx initiates second messenger activation, culminating in neuronal hyperexcitability (reviewed in Nassini et al., 2014). Additionally, Ca^2+^ and Ca^2+^-activated second messengers further regulate TRPA1 channel permeability by binding to intra- and extracellular Ca^2+^ binding domains (Doerner et al., 2007; Zurborg et al., 2007; Garrison and Stucky, 2011; Zhao et al., 2020). Ultimately, RAS and other noxious chemical compounds, which are produced exponentially following SCI, bind to cysteine residues on TRPA1 and activate the channel (Patapoutian et al., 2009; Garrison and Stucky, 2011; Materazzi et al., 2012; Toda et al., 2016), which elicit painful burning and prickling sensations characteristic of NP (Basbaum et al., 2009; Garrison and Stucky, 2011; Due et al., 2014; Park et al., 2015) and could be a contributory factor to hyperexcitable signaling underlying sensitization.

As previously mentioned, higher RAS levels have been associated with worse SCI and NP. Studies from our lab have demonstrated that significantly elevated acrolein levels corresponded with 2× upregulated TRPA1 mRNA levels in DRG alongside multimodal (mechanical, thermal, and chemical) sensory hypersensitivity and hyperalgesia in rat contusion and ischemic/reperfusion SCI paradigms (Due et al., 2014). Following SCI, TRPA1 mRNA was also found to be increased by 40× in peripheral nerve terminals and 2.5× in the spinal cord dorsal horn. Notably, TRPA1 mRNA upregulation was attenuated by administration of RAS-lowering drugs (Park et al., 2015). Acrolein injections to the spinal cord of naïve animals could reproduce similar behavioral hypersensitivity and cellular effects, including significant TRPA1 mRNA upregulation in DRGs, spinal cord dorsal horn, and peripheral nerve terminals (Park et al., 2015). Supporting these data are observations that acrolein administrated to extracted DRG cells *in vitro* resulted in cellular hyperactivity/sensitization, an effect that was exacerbated if the animal had experienced SCI before DRG extraction (Due et al., 2014). Interestingly, one study observed that SCINP was exacerbated via the upregulation and activation of TRPA1 through the exogenous inhalation of aerosolized acrolein and acrolein within cigarette smoke (Butler et al., 2017). The observation that exogenous inhaled acrolein worsens pain phenomena in SCI has also been observed clinically (Richards et al., 2005; Richardson et al., 2012).

Taken together, we hypothesize that chronic sensitization of the neuraxis, specifically marked by increased expression and sensitization of TRPA1 channels, may contribute to ongoing chronic neuropathic/neuroinflammatory pain even at normal systemic concentrations of RAS or during systemic perturbations such as acute illness or environmental exposure (diet or inhalation). Further work is required to characterize the relationship between RAS and inflammatory activation of TRPA1 in SCINP, but substantial preclinical evidence supports post-injury RAS-protein adduct upregulation and activation of TRPA1 leads to phenotypes consistent with SCINP.

#### Parallel inflammatory mechanisms

Chronic immune activation is implicated in a variety of NP disorders, including SCINP (Ellis and Bennett, 2013; Chen et al., 2016a; Ji et al., 2018). Neuroinflammation is involved in all parts of SCI secondary injury, which suggests persistent activation of peripheral immune and glial cells is vital for the development and maintenance of SCINP (Nesic et al., 2005; Hains and Waxman, 2006; Pineau and Lacroix, 2007; Detloff et al., 2008; Kim et al., 2009). The infiltration, retention, and chronic activation of these cells can influence local and remote environments through the release of pro-inflammatory signaling molecules (e.g., cytokines, chemokines, RAS, and ROS), which can in turn, modulate synaptic activity, leading to neuronal hyperexcitability. Hyperexcitability in nociceptive neurons (Bedi et al., 2010; Ritter et al., 2015) and dorsal horn superficial laminar neurons (Hains et al., 2003; Hains and Waxman, 2006; Latremoliere and Woolf, 2009) lead to spontaneous and hyperresponsive evoked pain in models of NP and SCINP (Kang et al., 2020). The increased input from hyperexcitable cells, alongside disrupted inhibitory input typically enacted via non-nociceptor gate-controlling sensory neurons and associated interneurons, results in increased transmission of nociceptive signals to the brain and heightened perception of pain (Baron, 2006).

Astrocytic and microglial reactivity correlate with increased spontaneous activity in nociceptors and hyperresponsiveness of dorsal horn neurons, resulting in sensitization to noxious stimuli alongside other electrophysiological and behavioral characteristics indicative of SCINP (Hains and Waxman, 2006; Carlton et al., 2009). Similar findings regarding peripheral immune cells have been extensively reviewed elsewhere (Ellis and Bennett, 2013; Finnerup et al., 2021). While neurons and glia are primarily implicated in the early neuroinflammatory response following SCI, peripheral immune cells are likely a critical driving force for the sustained and dysfunctional neuroimmune response in the chronic phases of secondary injury (Beck et al., 2010).

**Figure 4:**
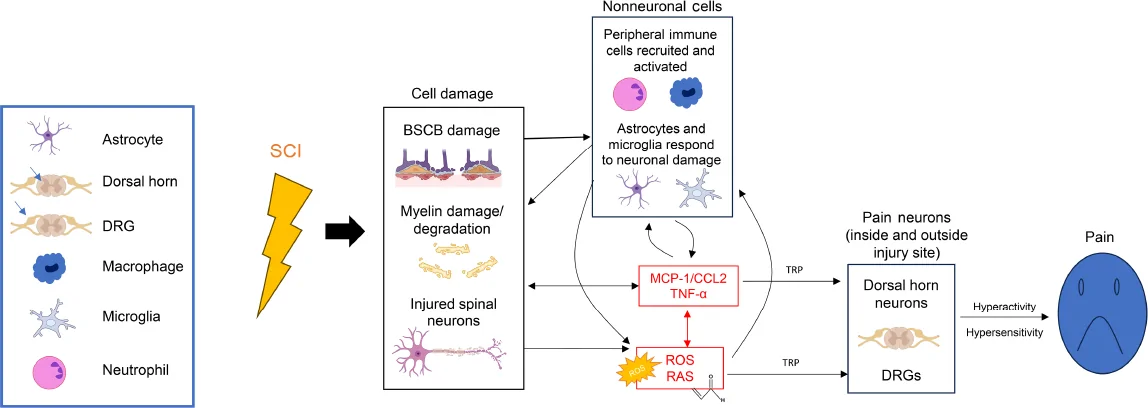
RAS and immune cells interact to incite spinal cord injury–related neuropathic pain. RAS/ROS recruits and retains immune cells in injured tissue, after which RAS/ROS and inflammatory cytokines (MCP-1/CCL2, and TNF-α) synergistically interact to incite hyperactivity and hypersensitivity in pain neurons. Created with BioRender.com. BSCB: Blood–spinal cord barrier; CCL2: C–C motif ligand 2; DRG: dorsal root ganglia; MCP-1: monocyte chemoattractant protein-1; RAS: reactive aldehyde species; ROS: reactive oxygen species; SCI: spinal cord injury; TNF-α: tumor necrosis factor α.

RAS can recruit immune cells and lead to their local retention in damaged or injured tissues. More specifically, RAS-protein adducts are ligands for macrophage scavenger receptors (Kirkham et al., 2003), which are involved in macrophage adhesion and retention (reviewed in Uchida, 1999). RAS-protein adducts can also directly activate macrophages, inducing the macrophage-driven release of ROS, monocyte chemoattractant protein-1 (MCP-1) (also known as C-C motif ligand 2 [CCL2]) (Kirkham et al., 2003), and tumor necrosis factor α (TNF-α) (Facchinetti et al., 2007). Isolated human neutrophils have also been found to produce RAS in ROS-induced inflammatory states (Anderson et al., 1997). Myelin damage also plays a role in macrophage activation, with RAS as a hypothesized mediator. Previous studies have implicated the role of RAS in myelin loss–induced axonal degeneration (Shi et al., 2011b; Ravera et al., 2015). The accumulation of myelin debris in the SCI epicenter induces foamy macrophage phenotype expression, which is long-lasting and pro-inflammatory (Greenhalgh and David, 2014; Wang et al., 2015; Zhu et al., 2017). These macrophages perpetuate the inflammatory and inhibitory nature of the SCI environment, amplify neurotoxicity, and thwart wound healing (Wang et al., 2015). They are also less efficient and effective at phagocytosis than microglia, more prone to apoptosis and necrosis after myelin phagocytosis, and have diminished capabilities to phagocytose apoptotic neutrophils. Un-phagocytosed neutrophils remain present and continue to release toxic contents, thus exacerbating ongoing tissue damage (Wang et al., 2015). Collectively, RAS can recruit and retain immune cells in injured tissue alongside induction and progressive accumulation of immune cells and pro-inflammatory molecule release (summarized in **Figure 4**).

Unsurprisingly, the continuous release of pro-inflammatory mediators, even at low levels, has been linked to NP (Martini and Toyka, 2004; Kohl et al., 2010). Such molecules can activate immune cells, modulate neuronal communication, induce changes in local and remote environments, initiate cell death, and activate intra- and extra-cellular signaling pathways (reviewed by Calvo and Bennett, 2012; Ellis and Bennett, 2013; Ji et al., 2013, 2016, 2018). Signaling molecule influences on neuronal cellular environments have been demonstrated using spinal nerve rhizotomy studies in rats. Researchers found evidence of sensitization, noting that uninjured neurons in the local environment exhibited hyperexcitable spontaneous activity and that this corresponded with behavioral hyperalgesia and allodynia (Wu et al., 2002; Ma et al., 2003). Because uninjured cell physiology was phenotypically consistent with NP, these findings suggest that the release of signaling molecules in response to injury can either change the local environment and/or directly activate neurons. Two studies utilizing rat T10 contusion SCINP models demonstrated that cytokine signaling could activate distant microglia, corresponding to behavioral phenotypes associated with SCINP (Detloff et al., 2008; Carlton et al., 2009). One study further observed that dorsal horn neurons at the same spinal level as activated microglia were hyperresponsive, which coincided with increased sensitization to mechanical and thermal stimuli (Carlton et al., 2009).

A prospective study in individuals with painful or painless neuropathy found pro-inflammatory cytokine (interleukin [IL]-2 and TNF-α) mRNA and protein levels to be significantly higher in patients with painful neuropathy compared to those with painless neuropathy and healthy controls. Interestingly, anti-inflammatory cytokine mRNA levels were found to be higher in patients with non-painful neuropathy compared to patients with painful neuropathy and healthy controls (IL-10: 2× higher than both groups; IL-4: 20× higher than painful neuropathy, 17× higher than healthy controls) (Sommer et al., 2018). Another study found that pro-inflammatory cytokines and other proteins (IL-6, IL-8, MCP-1/CCL2, tau, S100B, glial fibrillary acidic protein) from the cerebral spinal fluid of patients with SCI could predict the severity and onset of NP, AIS motor score, and functional improvements following their injury (Detloff et al., 2008; Kawasaki et al., 2008; Kwon et al., 2010, 2017). Taken together, these studies demonstrate that the presence of inflammatory molecules is correlated with NP severity and predicted outcomes and suggest that those with NP could have an imbalance of pro-/anti-inflammatory cytokine production (Sommer et al., 2018).

Chronic effects of pro-inflammatory signaling include the potentiation and maintenance of inflammation, increased nociceptor responsiveness, decreased nociceptive thresholds, and dysregulated neuronal plasticity (reviewed by Hucho and Levine, 2007; Basbaum et al., 2009). Among many important inflammatory mediators, MCP-1/CCL2 and TNF-α are notable for their extensively studied roles in neurodegenerative conditions and neuroinflammation. For example, TNF-α or MCP-1/CCL2 spinal cord injections in mice correspond with increased pain hypersensitivity, cytokine upregulation, mechanical allodynia, and heat hyperalgesia (Gao et al., 2009, 2010).

TNF-α is a cytokine that plays a vital role in inflammation and apoptosis. Following SCI, TNF-α is released by injured neurons and then by reactive microglia and peripheral macrophages as the secondary injury progresses (Shamash et al., 2002; Ellis and Bennett, 2013). One clinical study reported that TNF-α immunoreactivity correlated with axonal degeneration in patients with neuropathies (Lindenlaub and Sommer, 2003). TNF-α contributes to maladaptive nociceptive neuron and dorsal horn plasticity by increasing excitatory and decreasing inhibitory synaptic transmission, resulting in sensitization (Kawasaki et al., 2008). This finding was mechanistically supported by a study that observed application of TNF-α to peripheral nerve axons increased their firing rate (Sorkin et al., 1997). TNF-α can further modulate neuronal hypersensitivity and hyperactivity by stimulating the production and release of inflammatory signals such as MCP-1/CCL2 from astrocytes (Gao et al., 2009, 2010).

MCP-1/CCL2 is a chemokine known for recruiting immune cells and sustaining immune responses. It is produced by nociceptors, astrocytes, monocytes, and macrophages and binds to its receptor C–C Chemokine Receptor 2 (CCR2) (Jung et al., 2008; Gao et al., 2009, 2010), found in peripheral immune cells (Park et al., 2022) and neurons (Banisadr et al., 2005; Jung et al., 2008). MCP-1/CCL2 is upregulated during CNS inflammation and in DRGs following nerve injury (Jung et al., 2008; Gao et al., 2010; Roberts et al., 2012) and is linked to the development and severity of SCINP in patients. MCP-1/CCL2 can activate glial cells, recruit immune cells (Thacker et al., 2009; Pineau et al., 2010; Roberts et al., 2012; reviewed by Calvo and Bennett, 2012), and disrupt endothelial tight junctions in the blood–brain barrier, facilitating the entry of immune cells into the CNS (Roberts et al., 2012), and sensitizing nociceptive neurons (Jung et al., 2008). Additionally, MCP-1/CCL2 was found to indirectly induce axonal damage and degeneration through the activation of macrophages in an inflammatory NP model of Charcot-Marie-Tooth disease (Kohl et al., 2010). The binding of MCP-1/CCL2 to its receptor CCR2 has been shown to elicit neural sensitization and hyperexcitability, leading to the central transmission of nociceptive stimuli (Wu et al., 2002; Jung et al., 2008; Gao et al., 2009). CCR2 has been found in TRPV1^+^ and TRPA1^+^ nociceptive neurons, which also release MCP-1/CCL2 into the dorsal horn of the spinal cord (Jung et al., 2008; Van Steenwinckel et al., 2011). In a peripheral nerve injury model of NP, both damaged and neighboring undamaged sensory neurons released MCP-1/CCL2 from their central terminals into the CNS, which activated microglia (Thacker et al., 2009). These data suggest MCP-1/CCL2 signaling may be one of many potential mechanisms underpinning central sensitization via transference of inflammatory responses from peripheral to progressively central foci along the pain neuraxis.

As noted previously, 4-HNE and acrolein directly activate TRPA1 by binding to cysteine residues on its N-terminus (Cai et al., 2009) leading to the release of substance P and calcitonin-gene regulated-peptide into the spinal cord and subsequent neuronal hyperexcitability (Trevisani et al., 2007; Patwardhan et al., 2010). Direct activation of TRPA1 and synergistic actions with MCP-1/CCL2 are thought to be key mechanisms by which RAS can induce and perpetuate neuroinflammatory/NP states. TNF-α and MCP-1/CCL2 further modulate the activity of TRPA1 (Jung et al., 2008) and TRPV1 (reviewed in Ji et al., 2016), synergistically with the direct RAS-mediated activation. The upregulation of TRPA1 seen in states of NP could be in part related to TNF-α-mediated receptor recruitment to the cell membrane alongside enhanced Ca^2+^ influx (Meng et al., 2016; Silverman et al., 2020). Ca^2+^ influx has been observed to induce MCP-1/CCL2 release from TRPV1^+^ and TRPA1^+^ neurons into the dorsal horn (Jung et al., 2008; Van Steenwinckel et al., 2011), resulting in spinal microglial activation and production of NP-like behavioral hypersensitivity (Thacker et al., 2009). MCP-1/CCL2 can further synergize with acrolein/RAS leading to sensitization and upregulation/increased expression of TRPA1 (Jung et al., 2008). Collectively, the literature supports a plausible mechanistic relationship between RAS, oxidative stress, inflammatory mediator production, immunomodulation, and neuronal hyperactivity/sensitization via TRPA1 (reviewed in Stevens and Maier, 2008).

## Reactive Aldehyde Species as a Therapeutic Target in Spinal Cord Injury–related Neuropathic Pain

While the solution(s) for SCINP are currently unclear, it is apparent that existing tools fail to help many patients in need. Shortcomings in SCINP therapeutics can be attributed to numerous causes. First, the complexity of the underlying mechanisms between different disorders for which treatments have been approved for clinical use makes repurposing/off-label usage of existing therapeutics difficult and fraught with challenges (Teixeira-Santos et al., 2020). Current treatments merely modulate pain and suppress neuronal activity for a limited time, rather than correcting underlying causative mechanism(s) (Finnerup et al., 2021), which limits efficacy. Some treatments have produced modest effects, but the high doses required for efficacy bear detrimental side effects (Hurlbert, 2001; Hagen and Rekand, 2015; **Table 1**). Of additional concern are flaws in study design and data interpretation marring conclusions regarding efficacy, which may have contributed to the premature widespread use of ineffective medications with pronounced adverse effect profiles (Hurlbert, 2001; Chen et al., 2023). Taken together, new therapies must target the underlying mechanisms of SCINP while limiting side effects to optimize the odds of demonstrating clinical efficacy.

As detailed above in post-translational modification of pain-relevant membrane proteins, TRPA1, a nonselective cation channel in sensory neurons (Guimaraes and Jordt, 2007; Giorgi et al., 2019), has been identified as a key mediator of neurogenic inflammation and NP (Bautista et al., 2006; Trevisani et al., 2007; Logashina et al., 2019). We have outlined extensive evidence regarding RAS actions on TRPA1 (Guimaraes and Jordt, 2007; Giorgi et al., 2019), and in producing and sustaining NP in SCI and in other models of neuroinflammatory pain (Trevisani et al., 2007; Due et al., 2014; Park et al., 2015; Chen et al., 2016; Achanta and Jordt, 2017; Butler et al., 2017). Selective inhibition of TRPA1 or genetic knockout (TRPA1^–/–^ animals) produces analgesic effects (da Costa et al., 2010; del Camino et al., 2010; Vilceanu and Stucky, 2010; Chen et al., 2011; Wei et al., 2011; Luo et al., 2019) and has been recapitulated in humans where selective pharmacologic TRPA1 antagonism is analgesic (Heber et al., 2019). SCI increases TRPA1 expression 2–2.5× in the spinal cord dorsal horn and DRGs and 40× in peripheral nerves (Due et al., 2014; Park et al., 2015; Chen et al., 2016). Acrolein injections into uninjured spinal cords also elevate TRPA1 levels and mimic both behavioral and physiological DRG hypersensitivity that occurs following contusive SCI (Due et al., 2014; Gianaris et al., 2016). Excessive RAS concentrations upregulate, directly activate, and, through allosteric modulation, lower activation thresholds of TRPA1 (Achanta and Jordt, 2017). Collectively, TRPA1 changes following SCI are likely a critical contributor to underlying pain neuraxis hyperexcitability (Due et al., 2014; Park et al., 2015). Thus, we suggest the joint RAS/TRPA1 mechanistic pathway as a prime target for innovative therapeutics in SCINP, with possible extension of beneficial effects to other neuroinflammatory pain states.

A natural conclusion from the data presented would be to consider the development of selective TRPA1 inhibitors, which have already been pursued for other inflammatory pain conditions (Koivisto et al., 2024). Importantly, TRPA1 is essential for hearing (Corey et al., 2004; Nagata et al., 2005), airway protection (Lee, 2010; Chung et al., 2019; Xu et al., 2019), and in the GI tract (Csekő et al., 2019), raising concern for off-target effects in non-target tissue. Notably, TRPA1 programs by major pharmaceutical companies have failed in recent years due to inadequate safety/tolerability profiles in chronic animal administration studies or failure to meet primary efficacy endpoints during clinical trials (Jain et al., 2022; Bamps et al., 2023; Eli Lilly and Company, 2023; Koivisto et al., 2024). Despite this, direct TRPA1 modulators that are safe and tolerable could be developed eventually. One TRPA1 inhibitor, HC-030031 reduced mechanical and cold hyperalgesia and prevented upregulation of TRPA1 mRNA in the spinal cord and DRGs in chronic inflammatory (da Costa et al., 2010) and spinal nerve ligation (Eid et al., 2008) NP models without deleterious off-target effects. While direct inhibition of TRPA1 sounds appealing in SCINP, we anticipate this strategy may be limited by excess post-SCI RAS-mediated TRPA1 receptor upregulation and neuronal hypersensitivity. Unfettered post-SCI RAS elevations would act in direct opposition to TRPA1 antagonism, competing with the treatment itself. We suspect RAS elevations and actions on TRPA1 would necessitate higher doses of TRPA1 inhibitors for efficacy, increasing the risk of drug-related adverse effects. Furthermore, while we have focused on TRPA1 due to the depth of related evidence, we anticipate it is not the only relevant receptor involved in SCINP. Pain-relevant neuronal RAS protein targets include several other ion channels (e.g., NaV1.8, TRPV1, and CaVx), through which RAS can induce neuronal hyperactivity by post-translational modification or direct agonism (Bierhaus et al., 2012; Huang et al., 2016).

As noted previously, extensive preclinical evidence demonstrates that decreasing SCI secondary injury burden by reducing oxidative stress and inflammation can have beneficial effects on tissue and behavioral outcomes in SCI and SCINP (Luo and Shi, 2004; Bains and Hall, 2012; Hassler et al., 2014). Despite the critical pathological role of ROS, free radical scavengers and antioxidants have been largely unsuccessful in clinical trials (Hamann and Shi, 2009; Bains and Hall, 2012). Hypotheses to explain this include a short viable therapeutic window and high drug concentrations required to effectively capture ROS before they bind to biological targets due to their extremely short half-lives (Bains and Hall, 2012). RAS are attractive putative therapeutic targets because of their longer biologic half-lives compared to ROS and reactive nitrogen species, pro-inflammatory and pro-nociceptive characteristics, role in LPO and oxidative stress, and activation/upregulation/hypersensitization of TRPA1, among other pain-relevant receptors.

Inhibition of enzyme-mediated RAS production pathways (e.g., myeloperoxidase, polyamine oxidase, and spermine oxidase) is one attractive potential strategy. Unfortunately, there are at least five RAS production pathways relevant to SCI, which would require the co-administration of multiple enzyme inhibitors (Esterbauer et al., 1991; Stevens and Maier, 2008). In addition, these enzymes are critical for other important bodily functions and their inhibition is likely to have detrimental off-target effects. Boosting endogenous reactive aldehyde clearance mechanisms, namely via the ALDH superfamily modulation may be another approach (Bierhaus et al., 2012; Li et al., 2018c). More specifically, enhancing ALDH2 has been observed to combat oxidative stress-LPO-RAS-induced damage (Rodríguez-Zavala et al., 2019). Studies have demonstrated ALDH2 enhancement to be beneficial in a coronary artery disease ischemic-reperfusion injury with NP and an NP pain model related to DRG compression (Li et al., 2018a; Ding et al., 2019). Valuable insights have been gleaned from a transgenic mouse model of ALDH2*2, colloquially termed “Asian Flush Syndrome.” In this condition, ALDH2 is partially or almost fully inactive due to a point mutation responsible for encoding its catalytic site. These studies employed N-(1,3-benzodioxol-5-ylmethyl)-2,6-dichlorobenzamide (Alda-1), a novel ALDH2 activator molecule that acts as a molecular chaperone and restores the structural defect of the catalytically impaired enzyme (Chen et al., 2014; Rodríguez-Zavala et al., 2019). Alda-1 can do this by exerting allosteric properties, binding to the aldehyde binding site, and modifying its catalytic pocket. In turn, this restores the kinetic properties of ALDH2, improves its stability, and protects it from RAS-mediated inactivation (Chen et al., 2014; Rodríguez-Zavala et al., 2019).

We employed Alda-1 in an animal model of SCI and SCINP, finding that SCINP-like behaviors were chronically improved in animals receiving Alda-1. Notably, SCI acutely lowered the expression of ALDH2, which was preventable with Alda-1 administration, and enhanced ALDH2 activity via Alda-1 significantly lowered tissue acrolein and systemic acrolein metabolite levels (Herr et al., 2021). The upside of Alda-1 and similar ALDH-focused enhancers may be limited in SCI; high concentrations of RAS directly inhibit ALDH activity (Ren et al., 1999; Irwin et al., 2002; Herr et al., 2021), and thereby directly interfere with the primary mechanism of action. Furthermore, Alda-1 and other agents in its class suffer from short half-lives and poor pharmacokinetics, which in part contributed to the failure of Alda-1 in a phase I clinical trial for an unrelated condition (No author listed, 2011).

The limitations described above impact the feasibility of therapeutic RAS-lowering strategies focused on decreasing endogenous production or enhancing endogenous clearance. Direct RAS scavenging via nucleophilic compounds provides an alternative, where small molecules directly scavenge electrophilic RAS via 1:1 covalent binding. Three existing US Food and Drug Administration-approved medications are potent RAS-lowering drugs: hydralazine, phenelzine, and dimercaprol. Our lab has been instrumental in characterizing the benefits of hydralazine and phenelzine administration in SCINP animal models.

**Figure 5:**
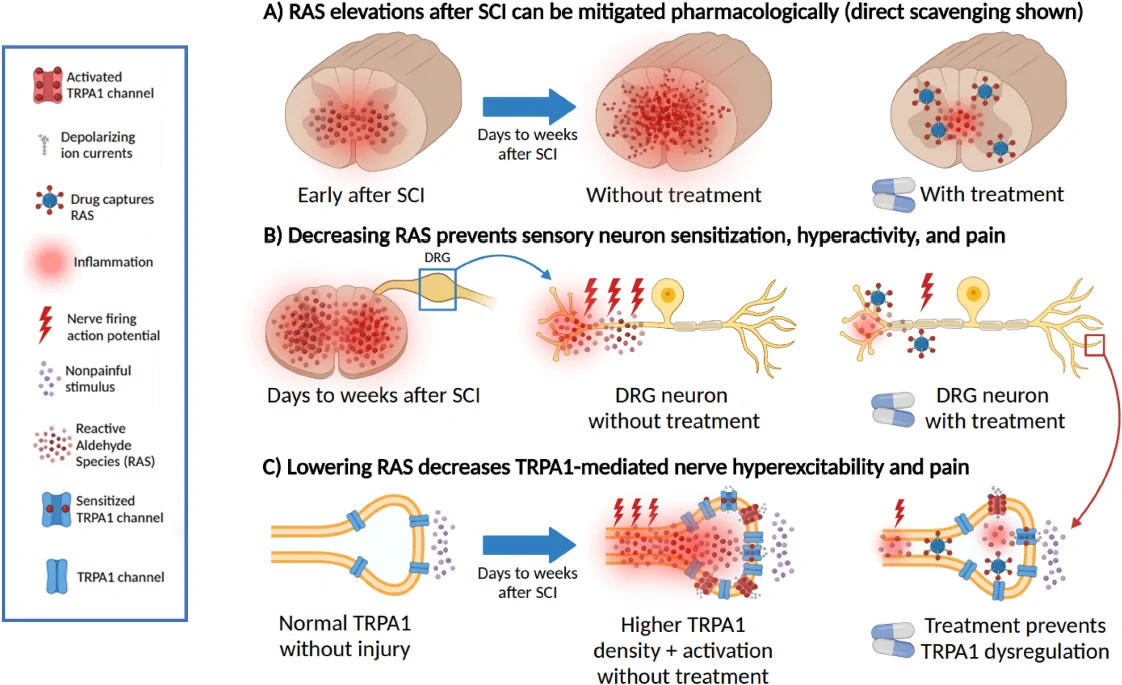
RAS and TRPA1 contribute to the development of spinal cord injury-related neuropathic pain in animal models. (A) RAS are profoundly elevated after SCI for days to weeks, an effect that can be mitigated with pharmacologic intervention. (B, C) RAS-induced upregulation, sensitization, and activation of TRPA1 in peripheral nerve terminals, DRG, and the dorsal horn leads to increased spontaneous neuronal firing and lower thresholds required to induce evoked firing (allodynia and hyperalgesia) and signal transmission to the brain, where the signals may be perceived as painful. Pharmacologic intervention can prevent these phenomena and reduce pain-related spontaneous and evoked signal transmission by sensory neurons. Created with BioRender.com. DRG: Dorsal root ganglia; RAS: reactive aldehyde species; SCI: spinal cord injury; TRPA1: TRP ankyrin 1.

Hydralazine is an anti-hypertensive vasodilator with a nucleophilic hydrazine functional group. The hydrazine group has similar properties to the nucleophilic centers of DNA and proteins (Shi et al., 2011) and can scavenge acrolein and related RAS through the formation of a 1:1 adduct via Michael addition (Burcham et al., 2000; Shi et al., 2011). Acrolein-hydralazine adduct formation reduces the toxicity of acrolein, thereby neutralizing it (Burcham et al., 2000, 2002, 2012; Burcham and Pyke, 2006), and the adduct can be safely eliminated from the body (reviewed by Park et al., 2014a). An *in vitro* RAS-induced biochemical injury to PC-12 cells was one of the first to demonstrate the neuroprotective capabilities of hydralazine. Here, hydralazine was shown to attenuate RAS-induced membrane damage, mitochondrial dysfunction, GSH depletion, and adenosine triphosphate reduction (Liu-Snyder et al., 2006). Next, an *ex vivo* spinal cord preparation with compression SCI was treated with hydralazine, which alleviated acrolein-induced superoxide production, GSH depletion, mitochondrial dysfunction, membrane degradation, and restored compound action potential production (Hamann et al., 2008a, b). Hydralazine was then evaluated in a rat T10 spinal cord contusion model. Excitingly, hydralazine could reach therapeutic concentrations in the spinal cord, decrease acrolein levels, and mitigate tissue damage, pain, and motor deficits (Park et al., 2014b). In subsequent studies, hydralazine treatment mitigated TRPA1 mRNA upregulation, SCINP-like behavioral hypersensitivity, and decreased excitotoxicity (Bai and Mei, 2011; Due et al., 2014; Park et al., 2014b; **Figure 5**). Importantly, hydralazine demonstrated the ability to longitudinally decrease behavioral hypersensitivity to mechanical and thermal stimuli (Due et al., 2014; Park et al., 2014b).

An alternative RAS scavenger to hydralazine is phenelzine, a monoamine oxidase inhibitor, which also possesses a nucleophilic hydrazine group. Like hydralazine, phenelzine scavenges acrolein in a 1:1 ratio and reduces acrolein levels following SCI (Hamann et al., 2008a; Hamann and Shi, 2009; **Figure 5**). The benefit of phenelzine is that it is not a vasodilator, so it can be used safely at higher concentrations than hydralazine (Hamann and Shi, 2009). Additionally, phenelzine has demonstrated the ability to scavenge RAS even after it has formed protein adducts (reviewed by Bains and Hall, 2012). In two *in vivo* rat studies – one using a T10 contusion model and one using an ischemic SCI model, phenelzine suppressed acrolein levels, reduced motor neuron loss, improved locomotor function and neuronal tissue preservation, attenuated NP behaviors (mechanical and thermal hypersensitivity), and decreased TRPA1 mRNA levels (Chen et al., 2016; Lin et al., 2018). Importantly, phenelzine attenuated SCINP behaviors in acute, delayed, and chronic administration in a dose-dependent manner, and symptoms notably returned or worsened after treatment was stopped (Chen et al., 2016).

Unfortunately, despite their positive attributes in lowering RAS and improving SCI and SCINP outcomes in animal models, hydralazine and phenelzine both have limitations precluding their candidacy for clinical translation. Hydralazine has a short half-life and a 1:1 acrolein scavenging ratio. As such, it may be difficult to achieve therapeutic levels clinically at tolerable doses (Park et al., 2014a). Tolerability is limited by the fact that hydralazine induces vasodilation as its primary mechanism of action, which is not ideal for SCI patients during hypotensive spinal shock or who chronically experience low blood pressure (Lehmann et al., 1987; Claydon and Krassioukov, 2006; Krassioukov et al., 2009; Shi et al., 2011; Park et al., 2014a). One proposed solution has been to implement di-hydralazine, which has two hydrazine groups (Kaminskas et al., 2004a; Shi et al., 2011).

Like hydralazine, phenelzine has attributes that limit its clinical translation. Monoamine oxidase inhibitor drugs are notorious for problematic, widespread drug-drug interactions and have potent psychotropic effects (Marley and Wozniak, 1984; Weideman et al., 1999; Gillman, 2011). Drug development professionals are also hesitant to pursue compounds with hydrazine groups, found in both hydralazine and phenelzine, due to case reports of auto-immunogenicity and hepatic injury, which are believed to be secondary to chronic accumulation of hydrazine-RAS-protein adducts that may become autoantigenic or potentially even toxic (Burcham et al., 2004; Kaminskas et al., 2004a). Hydrazines preferentially form adducts with and deactivate the carbonyl group, thus leaving the electrophilic β-carbon free to form drug–aldehyde–protein adducts, which have the potential to accumulate (Burcham et al., 2004; Kaminskas et al., 2004a). As such, hydrazines are typically unable to prevent cysteine-alkylation (β-carbon on RAS covalently binding to thiol groups on cysteine) (Burcham et al., 2004; Kaminskas et al., 2004b; Chavez et al., 2011). In theory, if two hydrazine-bearing molecules encountered “free” unbound RAS prior to reactive center contact with a protein moiety both reactive centers could be neutralized, but this seems unlikely due to relative local concentrations when compared to distribution volumes for systemic dosing.

Despite the limitations barring clinical translation of hydralazine and phenelzine, the efficiency of direct nucleophilic RAS scavenging by targeting the carbonyl group on α,β-unsaturated RAS is robust (Burcham et al., 2002; Kaminskas et al., 2004b; Burcham and Pyke, 2006; Song et al., 2010; Aldini et al., 2011; Colzani et al., 2016). Although hydrazine drugs do preferentially attack the carbonyl group on RAS, likely, the β-carbon of RAS has already adducted to another target, possibly before the hydrazine can even react with the RAS (Burcham et al., 2002; Kaminskas et al., 2004a). Thus, even suboptimal RAS scavengers have consistently demonstrated neuroprotective effects *in vitro*, *ex vivo*, and *in vivo* models of SCI and SCINP (Liu-Snyder et al., 2006; Hamann et al., 2008b, 2008a; Park et al., 2014b, 2015; Chen et al., 2016; Tian and Shi, 2017; Lin et al., 2018). Direct RAS scavengers benefit from not being tied to a singular RAS production or clearance pathway and effectively prevent/treat post-SCI sensory hypersensitivity in rats with low-dose daily intraperitoneal injections (Due et al., 2014; Park et al., 2014b, 2015; Chen et al., 2016; Lin et al., 2018). In SCI rats, acute treatment with RAS scavengers prevented SCINP in the immediate post-injury period and the delayed (subacute and chronic) post-injury period, even when dosing was delayed for weeks to months after injury (Due et al., 2014; Park et al., 2014b, 2015; Chen et al., 2016; Lin et al., 2018). RAS scavengers reduced paw withdrawal hypersensitivity (Due et al., 2014; Park et al., 2014b, 2015; Chen et al., 2016; Lin et al., 2018) and improved central affective measures of neuroinflammatory pain (Bai et al., 2012; Yao et al., 2017), both of which are mechanisms central to SCINP (Nesic et al., 2005; Hulsebosch, 2008; Knerlich-Lukoschus et al., 2008). Furthermore, RAS scavengers promote remyelination and neuroprotection, and improve locomotor recovery in animal models of SCI (Hamann et al., 2008b; Park et al., 2014b, 2015; Chen et al., 2016; Tian and Shi, 2017; Lin et al., 2018) with broader neuroprotective effects across other animal models of CNS trauma, ischemia, and degeneration (Wood et al., 2006; Leung et al., 2011; Musgrave et al., 2011; Singh et al., 2013; Shi et al., 2015; Cebak et al., 2017; Kulbe et al., 2018; Li et al., 2018b; Hill et al., 2019). Due to short drug half-lives and once daily dosing paradigms in the existing SCINP studies, it is possible and likely that positive effects of RAS scavengers could be further amplified by compounds with optimized dose-administration paradigms, improved pharmacokinetics, novel drug delivery strategies, and/or dual carbonyl and β-carbon inactivation to achieve cysteine thioprotection.

Dual carbonyl and β-carbon inactivation is theoretically achievable with small molecule technology, but limited candidate molecules have been exemplified in the existing literature. Histidine dipeptides such as carnosine and anserine have been published as a putative strategy with a two-step binding process, including initial reversible α,β-unsaturated imine formation followed by ring closure through an intra-molecular Michael addition observed *in vitro* (Aldini et al., 2002). Alternative observations such as the capability of edaravone to bind to RAS in a 2:1 ratio (2 edaravone molecules, 1 RAS molecule) to detoxify both reactive sites have been reported *in vitro* (Aldini et al., 2010). In both cases, it is unclear if the observed two-stage reactions would occur under *in vivo* conditions. Our lab attempted using dihydralazine to achieve this phenomenon *in vivo*, but it failed. More research is needed. Two-stage intra- or inter-molecular reactions facilitated by a single compound represent an approach that should be explored further with focused computer-aided drug design and medicinal chemistry optimization. Alternatively, joint delivery of distinct compounds optimized for detoxification of each site, the carbonyl and β-carbon, as combination therapy could be considered. Future studies in these areas will ideally include initial *in vivo* work to confirm reaction mechanisms are translatable to the *in vivo* microenvironment. We are aware of small molecule technology under development in both academic and industry settings reportedly capable of achieving two-step dual trapping. The details of these technologies have not been published to date and thus cannot be appropriately vetted for inclusion in this review beyond those highlighted above, but merit monitoring for further development.

Local, targeted drug delivery strategies could potentially circumvent the limitations for hydralazine, phenelzine, Alda-1, and even novel molecules that may face similar challenges. Targeted local drug delivery strategies aim to reduce the systemic burden of effective molecules that possess liabilities for prolonged systemic exposure (hydralazine, phenelzine), such that efficacy may be achieved with lower, focused dosing only at the site(s) needed. Nanoparticle-mediated focal drug delivery is one proposed strategy; preliminary data have been promising to utilize hydralazine-loaded chitosan nanoparticles (Cho et al., 2010) and mesoporous silica nanoparticles with encapsulated hydralazine (Cho et al., 2008; White-Schenk et al., 2013), but further work is needed to define optimal dosing, delivery strategies, and efficacy. Similarly, folate-conjugation linker strategies have recently shown promise in delivering hydralazine directly to SCI lesion sites in our animal models (Herr et al., 2022). This system, or a similar one, could also work for focused delivery of other compounds such as Alda-1. However, long-term safety data do not yet exist for these technologies.

## Biomarkers and Precision Medicine

As the next generation of RAS scavengers is developed, it is important to note that translational inter-species surveillance blood and urine biomarkers exist to support preclinical and clinical studies. Such biomarkers may be useful to prospectively stratify treatment responders (e.g., those with high RAS levels) and non-responders (e.g., those with lower RAS levels) and longitudinally monitor response to RAS-scavenging treatments. N-Acetyl-S-3-hydro-xypropylcysteine (3-HPMA) is a stable acrolein metabolite (1:1 reduction product of GSH and acrolein) found in serum and urine that historically has been utilized to monitor smoking status in human research subjects (Frigerio et al., 2020; Gallart-Mateu et al., 2024). 3-HPMA is most reliably quantified by liquid chromatography-tandem mass spectrometry, but more recently enzyme-linked immunosorbent assay and other, more accessible methods such as azide click reactions have also been developed (Higashi et al., 2016; Pradipta et al., 2016; Sakamoto et al., 2020). Assays measuring 3-HPMA have been implemented as a monitoring tool to trend systemic RAS levels longitudinally in animal and human studies of multiple sclerosis (Tully et al., 2018) and animal models of SCI (Zheng et al., 2013; Park et al., 2014). Research groups have also utilized 3-HPMA and enzyme-linked immunosorbent assays for serum levels of protein-bound acrolein in the development of multiplex diagnostic/prognostic assays for Alzheimer’s disease, ischemic stroke, end-stage renal disease, and Sjogren’s syndrome (Igarashi and Kashiwagi, 2011). Emerging methods of proteomic quantification of “RAS load,” the burden of RAS modification across the proteome, may become relevant in the future (Chen et al., 2019, 2021). While not yet available, imaging-based quantification of RAS would better enable longitudinal spatiotemporal mapping of RAS dynamics in SCI and other disease states. Imaging-based characterization of tissue/peri-lesional RAS burden would expand on the capabilities of blood- or urine-based biomarkers, enabling more precise approaches to disease trajectory and drug response monitoring.

## Conclusion

SCI is a devastating condition often made more complex by SCINP. SCINP is one of many sequelae of SCI that is worsened by post-injury biochemical and inflammatory cascades (secondary injury). Current SCINP treatments lack consistent efficacy and carry the risk of harm, making this area ripe for innovation. RAS and their established roles in SCI secondary injury, pain modulation (namely via TRPA1), and parallel neuroinflammatory pathways are convincing pathologic mediators in animal models of SCINP. RAS lowering strategies using US Food and Drug Administration-approved compounds for direct RAS scavenging or ALDH2 inhibitors have demonstrated significant potential to improve SCINP-relevant tissue, biochemical, physiologic, and behavioral outcomes in animal models. Further research into novel compounds with viable translational potential is warranted.

## Data Availability

*Not applicable.*
